# Lung ultrasound estimation of pleural effusion fluid and the importance of patient position

**DOI:** 10.1186/s13613-018-0471-x

**Published:** 2018-12-17

**Authors:** Luigi Vetrugno, Tiziana Bove

**Affiliations:** Division of Anesthesia and Intensive Care Medicine, Department of Medicine, University of Udine, P.Le S. Maria della Misericordia 15, 33100 Udine, Italy

Dear Editor,

We read with great interest the work by Razazi et al. [1] recently published in your estimated journal where the authors’ found an association between weaning failure and the interpleural distance in patients with difficult weaning. In agreement with the author, we reasoning that a successful pleural effusion (PLEFF) drainage is a fundamental component of the care we give to our patients [2]. For example, pleural drainage could be used to increase patient oxygenation in term of the ratio of partial pressure arterial oxygen and fraction of inspired oxygen (PiO_2_/FiO_2_) by re-expanding the collapsed lung with consequent benefits from mechanical ventilation interruption [3]. However, we would like to highlight some technical aspects in measuring PLEFF with ultrasound that deserve particular attention. The equation used by the author [1] and proposed by Balik et al. [4] is validated in mechanical ventilated patient in supine position and with a mild torso elevation of 15°, while the authors of the paper use a semi-recumbent position (i.e., head and torso at an angle of 40°–45°) [1]. This means that as fluid follow the law of gravity, an overestimation of the maximal distance between partial and visceral pleura could be obtained, and some examples are shown in Table 1. Furthermore, Balik’s equation [4, 5] overestimates in tall males with large thoracic circumference small effusions under 200 mL and in large ones above 1000 mL! And, the mean prediction error of this equation is quite high (158 ± 160 mL) [5] and although comparison of left and right side in terms of PLEFF, correlation did not show significant difference in the original study [4], other authors [6] have found a better correlation on the right side. In fact, the heart on the left increases the PLEFF like a stone in a water recipient leading to fluid overestimation. Consequently, we believe that the study of Razazi et al. [1] is up to date and very interesting, but an urgent standardization of the method to assess PLEFF with lung ultrasound is needed to reach a definite conclusion.

**Table 1:** An example of a possible relationship between the patient position and the estimation of volume of pleural fluid (mL) with maximum separation at lung base (Sep—mm), assuming an increase of 1 cm from A to B

		(A)		(B)
Balik formula [4]				
*V* (mL) = 20 × Sep (mm)		Supine position with a mild torso elevation of 15°		Semi-recumbent position (i.e., head and torso at an angle of 40°–45°)
Sep × 20	≥ 15 mm	300 mL (moderate)	25 mm	500 mL (large)
Sep × 20	≥ 25 mm	500 mL (large)	35 mm	700 mL (large?)

## Response

Keyvan Razazi^1,2,3^, MD, Armand Mekontso Dessap^1,2,3^, MD, PhD

^1^ AP-HP, Hôpitaux universitaires Henri Mondor, DHU A-TVB, Service de Réanimation Médicale, Créteil, 94010 France;

^2^ Université Paris Est Créteil, Faculté de Médecine de Créteil, IMRB, GRC CARMAS, Créteil, 94010, France.

^3^ Unité U955 (Institut Mondor de Recherche Biomédicale), INSERM, Créteil, France.

We thank Dr. Vetrugno and Dr. Bove for their interest and positive appreciations of our study “Pleural effusion during weaning from mechanical ventilation: a prospective observational multicenter study” [1]. Vetrugno and Bove highlight that pleural effusion was assessed supine with a mild torso elevation of 15° in the study by Balik et al. [4]. In our study, patients were in semi-recumbent position for ventilator-associated pneumonia prevention; this strategy often results in a median average elevations between 28.1° and 22.6° in clinical practice [7]. Although backrest elevation was not recorded in our study, we hypothesize that it was close to 25° in average and that pleural effusion might have been overestimated but only with a marginal effect. We agree that pleural effusion might be underestimated in tall men with Balik formula. We herein provide here the height of our patients, which was similar in patients with and without “moderate to large pleural effusion”: 169 ± 9 cm and 169 ± 10 cm, respectively. The association of pleural effusion with weaning failure persisted considering two others pleural effusion classifications: pleural effusion deemed drainable (as defined by a maximal interpleural distance ≥ 15 mm with the effusion visible over three intercostal spaces) or large pleural effusion with a maximal interpleural distance ≥ 25 mm. Vignon et al. showed a better correlation of volume of pleural with interpleural distance measured on the right side than on the left side and explained this difference by the presence of the heart. However, interpleural distance was measured in supine position in the study by Vignon et al., and this could have increased the “stone in a water effect” which was not found in the study by Balik et al. In our study, the maximal interpleural distance was equally located either on the left (*n* = 41, 51%) or right side (*n* = 40, 49%). We fully agree with the need for an urgent standardization of the method to assess pleural effusion volume. This point was not mentioned in the international recommendations of lung ultrasound [8].

## Abbreviations

PaO_2_/FiO_2_: the ratio of partial pressure arterial oxygen and fraction of inspired oxygen
PLEFF: pleural effusion
